# The molecular epidemiology of Foot-and-Mouth Disease virus serotypes A and O from 1998 to 2004 in Turkey

**DOI:** 10.1186/1746-6148-2-35

**Published:** 2006-12-04

**Authors:** Joern Klein, Ünal Parlak, Fuat Özyörük, Laurids S Christensen

**Affiliations:** 1 Danish Institute for Food and Veterinary Research, Department of Virology, Lindholm, 4771 Kalvehave, Denmark; 2 Foot-and-Mouth Disease Institute, Ankara, Turkey

## Abstract

**Background:**

Foot-and-Mouth Disease (FMD) causes significant economic losses in Turkish livestock.

We have analysed the genetic diversity of the 1D sequences, encoding the hypervariable surface protein VP1, of Turkish isolates of serotype A and O collected from 1998 to 2004 in order to obtain epidemiological and immunological information.

**Results:**

The 1D coding region of 33 serotype O and 20 serotype A isolates, obtained from outbreaks of FMD between 1998 and 2004, was sequenced.

For serotype A, we confirmed the occurrence of the two subtypes IRN99 and IRN96. These subtypes are most divergent within the region encoding the immuno-dominant GH-loop. Also a close relationship to Foot-and-Mouth Disease virus (FMDV) serotype A isolates obtained from outbreaks in Iraq and Iran were detected and a clustering of isolates collected during the same period of time were found.

The analysis of the deduced amino-acid sequences of these subtypes revealed evidence of positive selection in one site and one deletion, both within the GH-loop region. By inferring the ancestral history of the positively selected codon, two potential precursors were found. Furthermore, the structural alignment of IRN99 and IRN96 revealed differences between the tertiary structures of these subtypes.

The similarity plot of the serotype O isolates suggested a more homogeneous group than the serotype A isolates. However, phylogenetic analysis revealed two major groups, each further divided in subgroups, of which some only consisted of Turkish isolates.

Positively selected sites and structural differences of the Turkish isolates analysed, were not found.

**Conclusion:**

The sequence and structural analysis of the IRN99 strains is indicative of positive selection suggesting an immunological advantage compared to IRN96. However, results of antigenic comparison reported elsewhere do not substantiate such a conclusion. There is evidence that IRN99 was introduced to Turkey, in all probability from Iran.

Since, a member of the IRN96 lineage was included as a component of the FMDV vaccine produced since 2000, the outbreaks caused by IRN96 strains in 2004 could be due to incomplete vaccine coverage.

The Turkish type O strains, all with a VP1 structure similar to the O1/Manisa/69 vaccine, appear in several sublineages. Whether these sublineages reflect multiple samplings from a limited number of outbreaks, or if they reflect cross-boundary introductions is not clear.

## Background

Foot-and-Mouth Disease (FMD) is an acute, highly communicable and economically important disease caused by Foot-and-Mouth Disease virus (FMDV). Animals that can be affected include cattle, swine, sheep, goats, wild pigs, wild ruminants and buffaloes [1]. FMDV is a positive sense single-stranded RNA virus (genus *Aphthovirus*, family *Picornaviridae*) occurring in seven serotypes, O, A, Asia 1, C, SAT 1, SAT 2 and SAT 3, each with a wide spectrum of antigenic and epidemiological different subtypes. The wide diversity is considered a consequence of the high mutation rate and quasi-species dynamic [2].

FMD has been one of the most important diseases causing significant economic losses in the Turkish livestock sector. Together with production losses, export restrictions on several agricultural products cause additional losses to the Turkish economy [3].

Turkey is of special interest for the European countries, because it is the natural connection to Asia, where FMD is enzootic and thus serves as buffer zone against FMD [3].

The two prevalent serotypes in Turkey are A and O [4], and especially the pandemic FMDV serotype O is a major threat to Europe, because it is the most aggressive serotype [5]. However, this type O is still covered by the O1/Manisa/69 vaccine [4].

Serotype A displays a great antigenic diversity of subtypes and there is also often no cross-protection between them [5]. Since 1999 two subtypes of serotype A have been circulating in Turkey, designated by the World reference Laboratory IAH Pirbright as IRN96 and IRN99 [6].

Turkey practices vaccination against FMD, using a self produced trivalent vaccine consisting of O1/Manisa/69, Asia1 and since September 2000 a member of the IRN96 lineage as a replacement for A22, previously included [personal communication].

Due to the introduction of a twice-yearly national vaccination campaign in 1996, the number of FMD outbreaks has decreased significantly, but not homogeneously over the whole country, which may be explained by vaccine failure or incomplete vaccine coverage [7].

A common and major epitope of FMDV is located within the surface protein VP1, containing the immuno-dominant GH-loop and the RGD-integrin binding motif, essential for cell attachment [8]. Changes in this protein may cause vaccine failure and changes in host specificity [9].

We describe the genetic diversity of the 1D nucleotide sequence which encodes for the VP1 protein, in isolates obtained from outbreaks in Turkish cattle herds between 1998 and 2004 and analyse the data with regard to potential epidemiological information and to establish possibly whether FMD outbreaks are caused by viruses persistently circulating and evolving in Turkey or by strains introduced to Turkey.

## Results

### Phylogenetic inference

**Figure 1:**
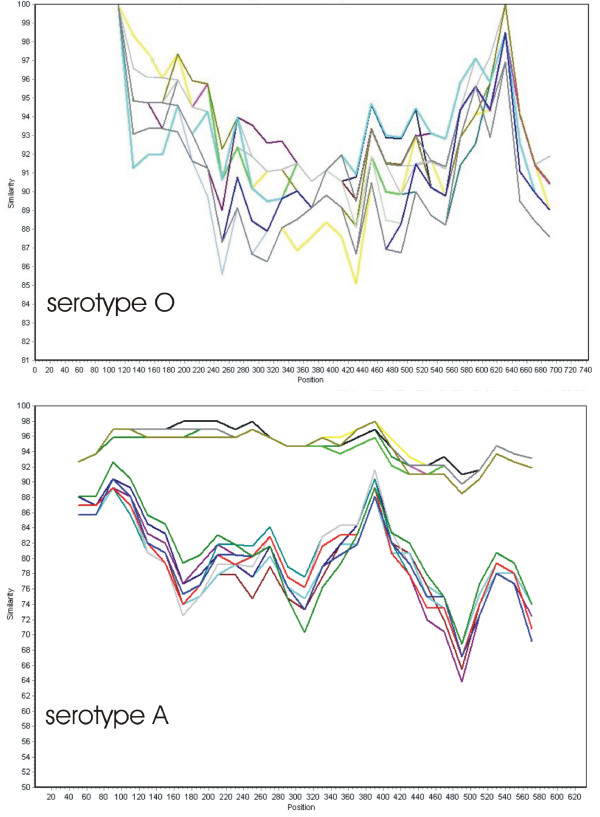
**Similarity plot of the 1D nucleotide sequence from the Turkish serotypes O (upper panel) and A (lower panel) obtained in this study**. Each coloured line indicates a single FMDV nucleotide sequence.

The 1D nucleotide sequence similarity scan (Figure 1) of the Turkish isolates of the present study reveals two well separated groups for serotype A. The two groups are most similar at position 90 nt with 8% divergence given a windows size of 20 nt and most divergent at position 490 with 31% divergence. The latter region encodes the immuno-dominant GH-loop. The diversity within each of the two serotype A groups is low within a range of 2–4%.

Serotype O, with O1/Manisa/69 as reference sequence, displays one homogeneous group, with a diversity in the range 9%.

**Figure 2:**
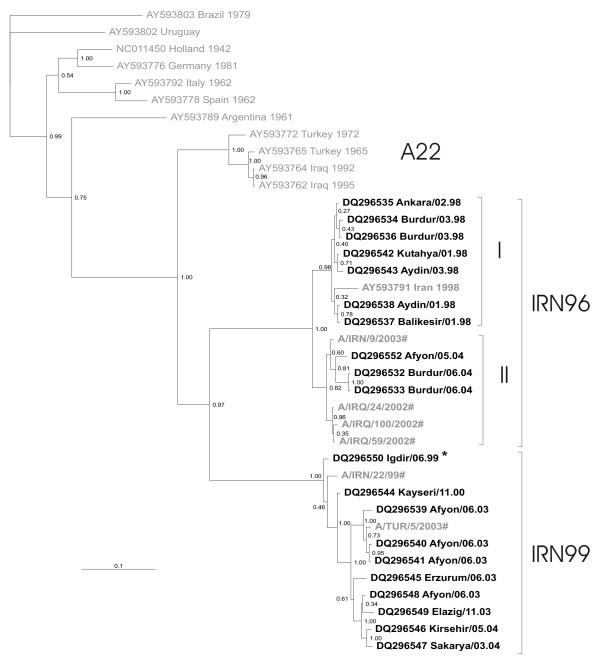
**Bayesian phylogenetic analysis of the 1D (VP1) nucleotide sequence of Turkish serotype A isolates (black) and related published sequences (grey)**. Numbers on the nodes indicate clade credibility values. Turkey related lineages are indicated by names given from the FMD World Reference Laboratory. The vaccine lineage (since 2000) IRN 96 is subdivided into two parts, linked to the year of collection. The first occurring isolate, within the IRN 99 cluster is indicated by an asterisk. Formats of sequence name: accession number-country/province-year/month, year of collection. Sequences ending with a '#' are kindly provided by IAH Pirbright.

The Bayesian tree, based on the 1D nucleotide sequence of serotype A isolates shown in Figure 2 reveals three clusters in Turkey, related to A22, IRN96 and IRN99. The most recent isolate of the A22 cluster, for which sequence information is available was collected in Iraq in 1995. The A22 cluster is distinctly separated from the IRN99 and IRN96 lineages as supported by a clade credibility value of 1.0 and the IRN96 and IRN99 clusters are separated as supported by a clade credibility value of 0.97.

The IRN96 cluster is further subdivided into two temporal divergent groups, I and II. The isolates of group I all originate from outbreaks in 1998, it was two years before this lineage was included in the Turkish FMD vaccine.

The IRN99 related viruses display a single, not further subdivided, phylogenetic structure; with the oldest isolate DQ296550 Igdir/06.99, obtained from an outbreak in June 1999 in the province Igdir, close to the Iranian border. Another member of IRN99 lineage is the isolate A/IRN/22/99# (sequence information kindly provided from IAH Pirbright) obtained from an outbreak in Iran in 1999.

**Figure 3:**
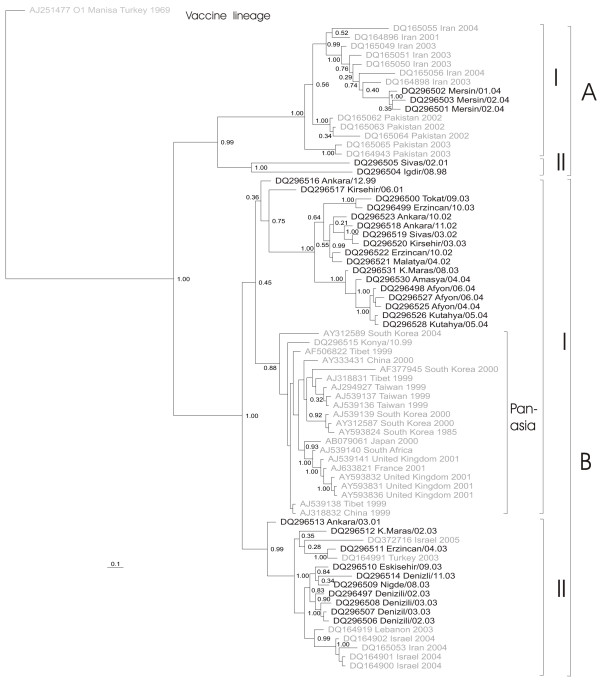
**Bayesian phylogenetic analysis of the 1D (VP1) nucleotide sequence of Turkish serotype O isolates (black) and related published sequences (grey)**. The vaccine strain O1 Manisa is used as outgroup. Numbers on the nodes indicate clade credibility values. Formats of sequence name: accession number-country/province-year/month, year of collection.

The inferred phylogeny of serotype O isolates (Figure 3), with the vaccine lineage as outgroup, displayed two major clusters A and B, supported by a clade credibility value of 1.0 (Figure 3).

Cluster A is split into the two groups I and II. Group I consists of sequences derived from FMD outbreaks in Pakistan and Iran in addition to a single outbreak in January/February 2004 in the Mersin region of Turkey. The differential success, expressed as correlation to the number of substitutions, with regard to the vaccine lineage, is lowest in the group members obtained from outbreaks in Pakistan in 2002 and 2003, intermediate in the Iranian isolates from 2003 and 2004 and most advanced in the Turkish isolates from 2004.

Group II of cluster A consists of Turkish isolates obtained from outbreaks in the provinces Sivas in 2001 and Igdir in 1998. The latter province is close to the Iranian border.

The majority of Turkish isolates belong to cluster B, which can also be subdivided into two groups I and II.

Cluster I of group B was further subdivided into two lineages. One group consists of isolates, obtained only from outbreaks in Turkey and the other of isolates known as the Panasia lineage. This partition is supported by a clade credibility value of 0.45. The Turkish lineage displays an increasing differential success rate, with regard to the vaccine strain O1/Manisa/69, starting from the DQ296516 Ankara/12.99 isolate, obtained in the Ankara province in 1999, towards the DQ296528 Kutahya/05.04 isolate, obtained in the Kutahya province in 2004.

Cluster II of group B consists of viruses obtained from outbreaks in Turkey, Israel and Lebanon, with the earliest obtained from an outbreak in the Turkish Ankara region in 2001.

### Positive selection analysis

**Figure 4:**
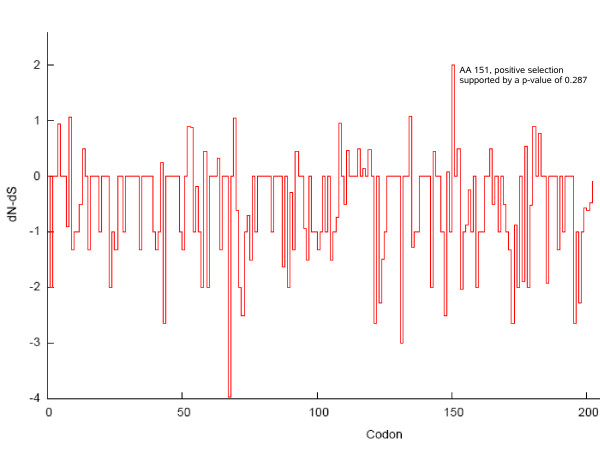
**Sliding window representing the rate of synonymous and nonsynonymus nucleotide substitutions per site from serotype A sequences**. The extent of positive and negative selection on individual codons. The value above and below the abscissa, indicates the number of per-site nonsynonymus substitutions (*d*_N_) over per-site synonymous ones (*d*_S_) in an individual codon. The zero value means *d*_N _= *d*_S_. Site 151 shows evidence for significant positive selection (*P *< 0.05) based on the extended binomial distribution.

The examination of the deduced VP1 amino acid sequences of the serotype O isolates for selective pressures, show no significant (defined as p > 0.5) evidence for positive selection (data not shown). On the contrary, residue 151 of serotype A isolates displays significant evidence (p = 0.29) for positive selection (Figure 4 and additional file 1). Residue 151 is located in the immuno-dominant GH-loop of the VP1 molecule and therefore particularly exposed to the immune system of the host.

By investigating the inferred ancestor history of site 151 (additional file 2 and 3), it can be seen that all but two of the IRN96 isolates, DQ296532 and DQ296533, each collected from the same outbreak, have the common ancestor codon 'AGC' (Ser), whereas IRN99 has most likely the 'GGG' (Gly) codon as precursor.

**Figure 5:**
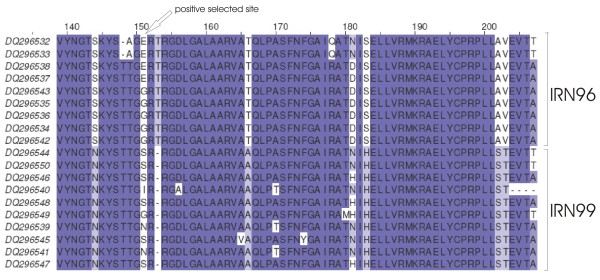
**Amino Acid – alignment of the GH-loop (residues 140–160) from both serotype A lineages IRN99 and IRN96**. Virus isolates are named after the Genbank Accession-number. Complete deletion at site 153 of the IRN99 lineage, as well as lineage specific conserved amino acids at the sites: 144, 166, 183, 202 and 203. The potential integrin binding site Arg-Gly-Asp (RGD) [8], is in all but DQ296541, as 'RGDLGAL'-motif present.

Comparing the translated amino acid sequences of the whole GH-loop region, located within the residues 140–160 (Figure 5), reveals that IRN99 isolates consistently deviates from IRN96 isolates by a deletion at site 153 as well as in lineage specific conserved amino acids at the sites: 144, 166, 183, 202 and 203. The integrin-binding site Arg-Gly-Asp (RGD) [8], was in all serotype A isolates, but DQ296540, as 'RGDLGAL'-motif present.

### Structural analysis

**Figure 6:**
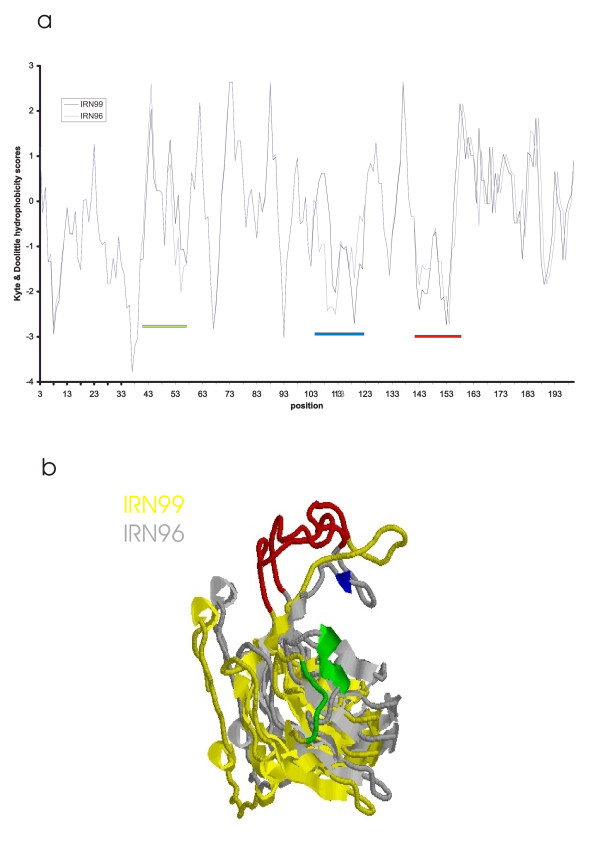
**Amino Acid Hydrophobicity plot (a) and structural alignment (b) based on the consensus sequences of the both serotype A lineages IRN99 andIRN96**. Significant differences are indicated by coloured bar, where the colour refers to the part of the structural model.

**Figure 7:**
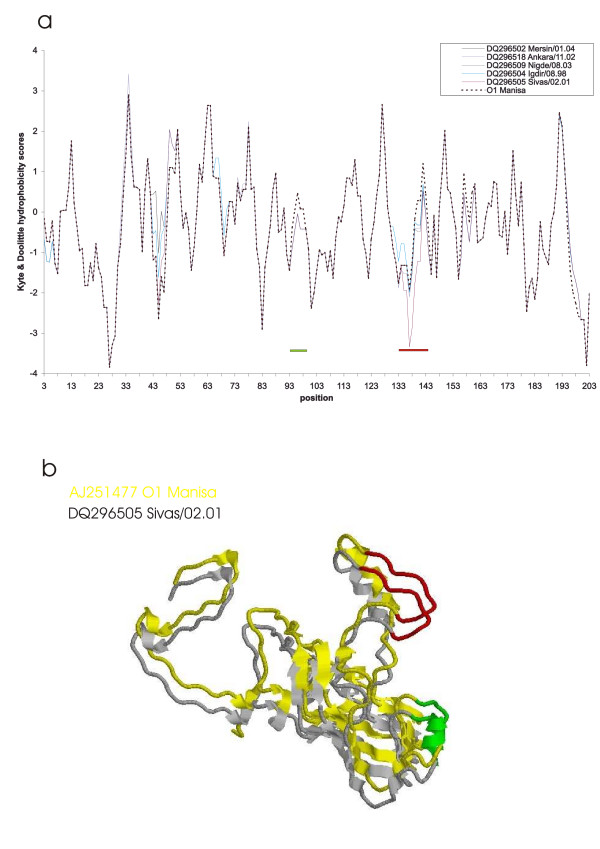
**Amino Acid Hydrophobicity plot (a) and structural alignment (b) based on the consensus sequences of the main serotype O subtypes**. Significant differences are indicated by coloured bar, where the colour refers to the part of the structural model.

To compare the VP1 protein secondary structure we analysed the putative hydrophobic and hydrophilic regions along the length of the VP1 amino acid sequence, using the Kyte and Doolittle algorithm [10]. This revealed three different regions for the consensus sequences of the IRN99 and IRN96 lineages (Figure 6a), as well as two major disparate regions for representatives of the serotype O lineages (Figure 7a). The structural alignment of the tertiary structure, based on the consensus sequences of IRN99 and IRN96 (Figure 6b), shown 75% sequence identity, revealed appreciable structural differences between the lineages, within the regions indicated by the hydrophobicity plot. There is evidence that the IRN96 lineage has two β-sheet structures at the amino acid positions 40–55 and 105–124, where IRN99 has none, as well as a different helix-structure at position 140–159 (Figure 6b).

From the hydrophobicity plot, DQ296505 Sivas/02.01 seems to represent the most distinct lineage of the Turkish serotype O isolates (Figure 7a). However, the structural alignment between DQ296505 Sivas/02.01 and the vaccine strain O1/Manisa/69, displays a high similarity of 99% (Figure 7b).

## Discussion

The similarity plot (Figure 1), as well as the phylogenetic analysis of serotype A (Figures 2), confirms the presence of two independent lineages within our collection of isolates. A member of the IRN96 cluster was not included in vaccines before September 2000, which means that the vaccines before that did not provide sufficient protection against IRN96 strains. However, the IRN96 isolates (Figure 2, IRN96 II) collected from outbreaks in 2004, and the clustering of these isolates with the IRN96 isolates from Iran and Iraq alternatively suggests multiple introductions from one or both of these countries.

The IRN99 lineage is suggested to be a newly introduced lineage rather than an escape mutant lineage evolved from IRN96. This is supported by the lack of evolutionary intermediates between the two clusters, the consistent differences between isolates of the two clusters and the low probability of a common ancestor of residue 151 potentially subject to positive selection. In addition that the first IRN99 isolate reported in Turkey originate from an outbreak in the province of Igdir, close to the Iranian border. The structural alignment of IRN99 and IRN96 (Figure 6) revealed significant conformational differences within this important immuno-dominant protein suggesting an immunological advantage of IRN 99 strains given that a member of the IRN96 is used as a vaccine component. However, antigenic comparisons based on virus neutralisation analysis and epidemiological data previously reported [12] do not substantiate such a conclusion.

Recalling the fact that structures are more conserved in evolution than sequences, these observed differences support the idea of an independently evolved IRN99 lineage. Recombination events between the two serotype A types IRN99 and IRN96 within the 1D region were not detected by the similarity scan. This may indicate that both serotypes A types are in the phase of high fitness and have found their particular ecological niche.

In contrast to the serotype A strains, the serotype O isolates included in this study constitute a more homogeneous group, as shown by the similarity plot (Figure 1).

The phylogenetic analysis of serotype O isolates demonstrate the presence of two major subtypes, cluster A and B (Figure 3), each further subdivided in distinct lineages. Cluster A I contained isolates from one single outbreak in the Turkish Mersin province, as well as isolates obtained from earlier outbreaks in Pakistan and Iran. The temporal distribution and the differential success of these isolates, i.e. the branch lengths, suggest that the virus was introduced from Pakistan to Iran and then to Turkey. Cluster A II, consisting of the Turkish isolates DQ296505 Sivas/02.01 and DQ296504 Igdir/08.98, is well separated from A I and represent a sublineage only observed in Turkey. The same conclusion may be valid for the cluster B I, which also exclusively comprised Turkish isolates. However, the intermediate clade credibility value of 0.45 at the node between cluster B I and the Panasia lineage may indicate a closer relationship to the pandemic Panasia lineage than displayed in the phylogenetic tree. An explanation for these purely Turkish sublineages may be that the virus is circulating in Turkish cattle herds, either through subclinically infected cattle or by transmission to cattle from other reservoirs such as sheep and goats.

Cluster B II is clearly separated from the Panasia lineage, consisting of isolates from newer outbreaks in Turkey, Israel and Lebanon, indicating the spread of this sublineage.

Nevertheless, the structural comparison between the field isolates and the vaccine strain O1/Manisa/69 (Figure 8) displayed no distinct differences suggesting that the vaccine strain, O1/Manissa/69, in current use should provide full protection. This suggestion is concordant with other studies [11,12].

## Conclusion

In conclusion, this study confirmed the presence of two major serotype A lineages, namely IRN 99 and IRN96, in Turkey. There was no evidence that the A22 strain was present in Turkey during the examined period of time, however this may be related to the small sample size used in this study. The sequence and structural analysis of the IRN99 related viruses revealed potential conformational differences of the IRN99 VP1 possibly affecting the antigenic properties of particular regions. There is evidence that IRN99 is introduced from outside to Turkey, in all probability from Iran.

Since, the IRN96 lineage is a component of the FMDV vaccine used since 2000, the outbreaks in 2004 occurred most probably due to incomplete vaccine coverage.

The endemic, slowly evolving Turkish O lineages, with a predicted VP1 structure similar to the O1 Manisa vaccine, occurs in several sublineages in Turkey. Some of these sublineages are most probably introduced from neighbouring countries, whereas others are circulating within Turkish cattle or are frequently reintroduced to cattle from persistently infected small ruminants, i.e. sheep and goats, which share pastures and transport vehicles.

## Methods

### Virus isolates

Thirty-three serotype O (Accession numbers: DQ296497 – DQ296531) and twenty serotype A (Accession numbers: DQ296532 – DQ296552) field isolates were collected from 1998 to 2004 from bovine herds widely distributed in Turkey. All isolates were epidermal tissue samples representing vesicular lesions and had been stored in TRIZOL^©^-reagent. Province and time (month/year) of collection are indicated with the accession numbers.

### RNA extraction, reverse transcriptase – PCR and cycle sequencing

Tissue (100–150 mg) was homogenized in 1 ml RNA*pro*™ Solution (Qbiogene, USA) in a Lysing Matrix D tube (Qbiogene, Inc., USA) using a FP 120 Fast Prep™ Cell Disruptor (Qbiogene, USA). Total RNA was extracted using RNeasy-Mini Kit™ (Qiagen, Germany) according to manufacturer's instructions. cDNA synthesis was done using

Ready-To-Go™ You-Prime First-Strand Beads (GE Healthcare Life Sciences, Sweden), employing the primers NV27T and random hexamers pdN_6_.

Five μl of the template cDNA were added to 45 μl of the PCR reaction mixture containing 0,2 μM primers (see Table 1), 200 μM each of dATP, dCTP, dGTP and dTTP, 10 mM Tris-HCl (pH 8.3), 50 mM KCl, 1.5 mM MgCl_2 _and 1 U of AmpliTaq^® ^Gold DNA polymerase (Applied Biosystems, UK). DNA was amplified with a DNA Thermal Cycler PE9700 (Perkin Elmer) by a two-step cycling reaction as follows: 95°C for 15 min, and five cycles of 94°C for 30 sec, 57°C for 2 min and 72°C for 30 sec, and then 35 cycles of 94°C for 30 sec, 61°C for 30 sec and 72°C for 30 sec, followed by a final extension step of 72°C for 10 min.

The resulting PCR product was examined by electrophoresis, using a 1,2% agarose gel, containing 0,005% ethidium bromide, with a separation time of 1,5 hours at 6,5 V/cm.

Amplicons were extracted and purified from agarosegel with QIAquick Gel Extraction kit (Qiagen, Germany) and cycle-sequencing was then performed by Agowa GmbH, Germany.

### Multiple alignment

Sequence assembling was performed with ContigExpress (VectorNTI^©^-software) and multiple alignment was performed by log-expectation comparison, using the MUSCLE (v.3.6) software [13]. For the serotype A alignment manual editing at codon position 153 was done.

### Similarity analysis

Similarity plots were performed using Simplot software [14] with the following parameters: For serotype A a windows size of 100 bp (step: 20 bp) with gap-stripping and Jukes-cantor correction was chosen, whereas a window of 200 bp (step:20 bp) with gap-stripping and Kimura (2-parameter) correction was chosen for serotype O.

### Phylogenetic analysis

Models of evolution were determined by hierarchical Likelihood-Ratio test of 24 substitution models, using the programs PAUP*(v. 10) [15] and MrModeltest (v. 2.2) [16].

For serotype A the HKY+G was used and Bayesian analysis was performed using MrBayes (v3.2) [17] with the following settings. The maximum likelihood model employed 2 substitution types ("nst = 2"), with base frequencies set to fixed values ("statefreqpr = fixed"). Rate variation across sites was modelled using a gamma distribution (rates = "gamma"). The Markov chain Monte Carlo search was run with 4 chains for 500000 generations, with trees begin sampled every 100 generations (the first 1000 trees were discarded as "burnin").

For serotype O the GTR+G was used and Bayesian analysis was performed using MrBayes (v3.2) [17] with the following settings. The maximum likelihood model employed 6 substitution types ("nst = 6"), with base frequencies set to variable values ("statefreqpr = dirichlet(1,1,1,1)"). Rate variation across sites was modelled using a gamma distribution (rates = "invgamma"). The Markov chain Monte Carlo search was run with 4 chains for 500000 generations, with trees begin sampled every 100 generations (the first 1000 trees were discarded as "burnin").

### Selection pressure analysis

Positively selected sites (Codon-specific analyses of dN/dS) was identified using the Single Likelihood Ancestor Counting (SLAC) analysis [18], a modification of the Suzuki-Gojobori method [19], available at the Datamonkey web side [18]. For both serotypes, the analysis was done with the HKY85 substitution model on phylogenetic trees inferred using the Neighbour-Joining method with a cut-off p value of 0.5.

### Structural analysis

Three-dimensional structure of the VP1 protein was obtained using comparative protein modelling [20], employing the CPHmodels 2.0 Server [21] and pairwise structure comparison was done using the DaliLite workbench [22]. Molecule Visualization was done using RASMOL 2.7 software [23].

The structural analysis of the A serotype is based on the comparison of the translated amino acid consensus sequences from the IRN96 and IRN99 lineage. Both, IRN99 consensus and IRN96 consensus sequences, used the structure of serotype A-1061 (1ZBA:1; Resolution: 2 Å) [24] as template.

For serotype O viruses the structural analysis was based on the translated amino acid sequences of DQ296505 Sivas/02.01 and the vaccine strain O1/Manisa/69. Both used the structure of strain O-1860 (1FOD:1; Resolution: 2,6 Å) [25] as template.

## Authors' contributions

JK carried out the molecular epidemiological studies, the sequence and structure analysis and drafted the manuscript. ÜP and FÖ collected the field isolates. LSC conceived the study, and participated in its design and coordination and helped to draft the manuscript. All authors read and approved the final manuscript.

## Supplementary Material

Additional file 1 SLAC and REL results Click here for file

Additional file 2 **Ancestral State Reconstruction of the codon sequence of site 151 mapped to a phylogeny**. Using the Maximum Likelihood the codons ancestral sequence is determined. The arrow indicates the changeover between the both lineages IRN99 and IRN96. Click here for file

Additional file 3 **Ancestral State Reconstruction of the amino acid of site 151 mapped to a phylogeny**. Using the Maximum Likelihood the codons ancestral sequence is determined. The arrow indicates the changeover between the both lineages IRN99 and IRN96. Click here for file

## Figures and Tables

**Table 1:** Primers used in this study

**Sense/Antisense**	**Designation**	**Sequence (5' to 3')**	**Published by**
Sense	O-1C244F	GCAGCAAAACACATGTCAAACACCTT	[26]
Sense	O-1C272F	TBGCRGGNCTYGCCCAGTACTAC	[26]
Sense	O-1C283F	GCCCAGTACTACACACAGTACAG	[26]
Antisense	NK61	GACATGTCCTCCTGCATCTG	[26]
Sense	SV1	GCGCCACACCGCGTGTTGG	[27]
Antisense	P1	GAAGGGCCCAGGGTTGGACTC	[27]
Sense	P498¶	AATTACACATGGCAAGGCCGACGG	[28]
Antisense	P617 rc	CCCGAGTGGCTTTGATGG	[28]
Sense	A1	ACCAATGTACAGGGATGGGT	This study
Antisense	A2	TTGCACCGTAGTTAAAGGAA	This study
Sense	B1	CTGAGAGAGCTGCACACTGC	This study
Antisense	B2	GTACTTGCTCGTCCCGTTGT	This study
