# Supplementary material for: The molecular epidemiology of Foot-and-Mouth Disease virus serotypes A and O from 1998 to 2004 in Turkey
Source: BMC Vet Res. 2006 Dec 4;2:35. doi: 10.1186/1746-6148-2-35 (PMC1698480; doi:10.1186/1746-6148-2-35)
Supplement: Additional file 1 — SLAC and REL results [file 1746-6148-2-35-S1.pdf]

# SLAC Analysis Results

**Nucleotide Model(010010) Fit Results**

Log likelihood = -1787.09078680109

Nucleotide substitution rate matrix

| | A | C | G | T |
|---|---|---|---|---|
| **A** | * | 0.162401 | 1 | 0.162401 |
| **C** | - | * | 0.162401 | 1 |
| **G** | - | - | * | 0.162401 |
| **T** | - | - | - | * |

**Codon Model Fit Results**

Log Likelihood = -1705.12560615312

Tree length (expected substitutions per site) = 0.341605

Global dN/dS = 0.167436

FOUND **1** POSITIVELY SELECTED SITES (0.3 significance level )

| Codon | dN-dS | Normalized dN-dS | p-value |
|---|---|---|---|
| 151 | 2.00565 | 2.36703 | 0.287108 |

# REL Analysis Results

**Codon Model(010010) Fit Results**

Log likelihood = -1678.99, AIC = 3453.97

Nucleotide substitution rate matrix

| | A | C | G | T |
|---|---|---|---|---|
| **A** | * | 0.206492 | 1 | 0.175733 |
| **C** | - | * | 0.0804541 | 0.694633 |
| **G** | - | - | * | 0.204151 |
| **T** | - | - | - | * |

Total Tree Length in E[subs/site]:0.388528

**Fitted distributions.**

| Synonymous (dS) | | Non-synonymous (dN) | | Non-syn and syn difference (dN-dS) | |
|---|---|---|---|---|---|
| **Rate** | **Weight** | **Rate** | **Weight** | **Rate** | **Weight** |
| 0.00000000 | 0.0004947 | 0.00296214 | 0.6655950 | -1.09329965 | 0.6071497 |
| 0.00000000 | 0.0873145 | 0.40204362 | 0.3187059 | -0.69421817 | 0.2907206 |
| 1.09626179 | 0.9121909 | 3.35760707 | 0.0156078 | 0.00296214 | 0.0584453 |
| | | 10.26474971 | 0.0000913 | 0.40204362 | 0.0279853 |
| | | | | 2.26134528 | 0.0142373 |
| | | | | 3.35760707 | 0.0013705 |
| | | | | 9.16848792 | 0.0000833 |
| | | | | 10.26474971 | 0.0000080 |
| Mean = **1**<br>Variance = **0.0962618** | | Mean = **0.183447**<br>Variance = **0.203445** | | Mean = **-0.816553**<br>Variance = **0.299707** | |

FOUND **2** POSITIVELY SELECTED SITES (50 significance level )

| Codon | E[dS] | E[dN] | Normalized E[dN-dS] | Posterior{dN>dS} | Bayes Factor{dN>dS} |
|---|---|---|---|---|---|
| 9 | 1.08312 | 3.36074 | 5.86219 | 0.980094 | 432.862 |
| 151 | 1.01061 | 3.2524 | 5.76997 | 0.96414 | 236.372 |
