# Supplementary figures and images for: The molecular epidemiology of Foot-and-Mouth Disease virus serotypes A and O from 1998 to 2004 in Turkey
Source: BMC Vet Res. 2006 Dec 4;2:35. doi: 10.1186/1746-6148-2-35 (PMC1698480; doi:10.1186/1746-6148-2-35)

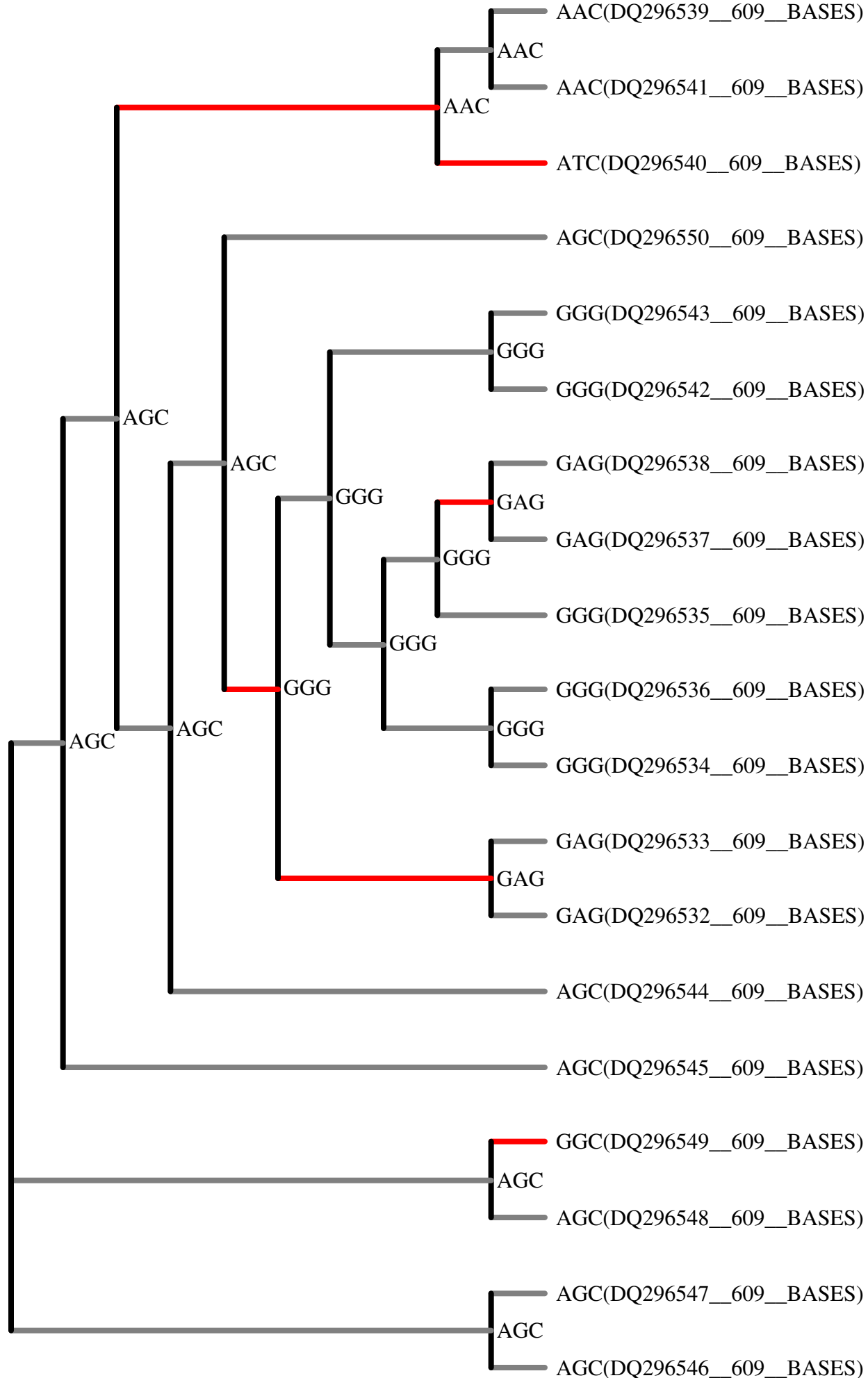

Supplement: Additional file 2 — Ancestral State Reconstruction of the codon sequence of site 151 mapped to a phylogeny. Using the Maximum Likelihood the codons ancestral sequence is determined. The arrow indicates the changeover between the both lineages IRN99 and IRN96. [file 1746-6148-2-35-S2.pdf]

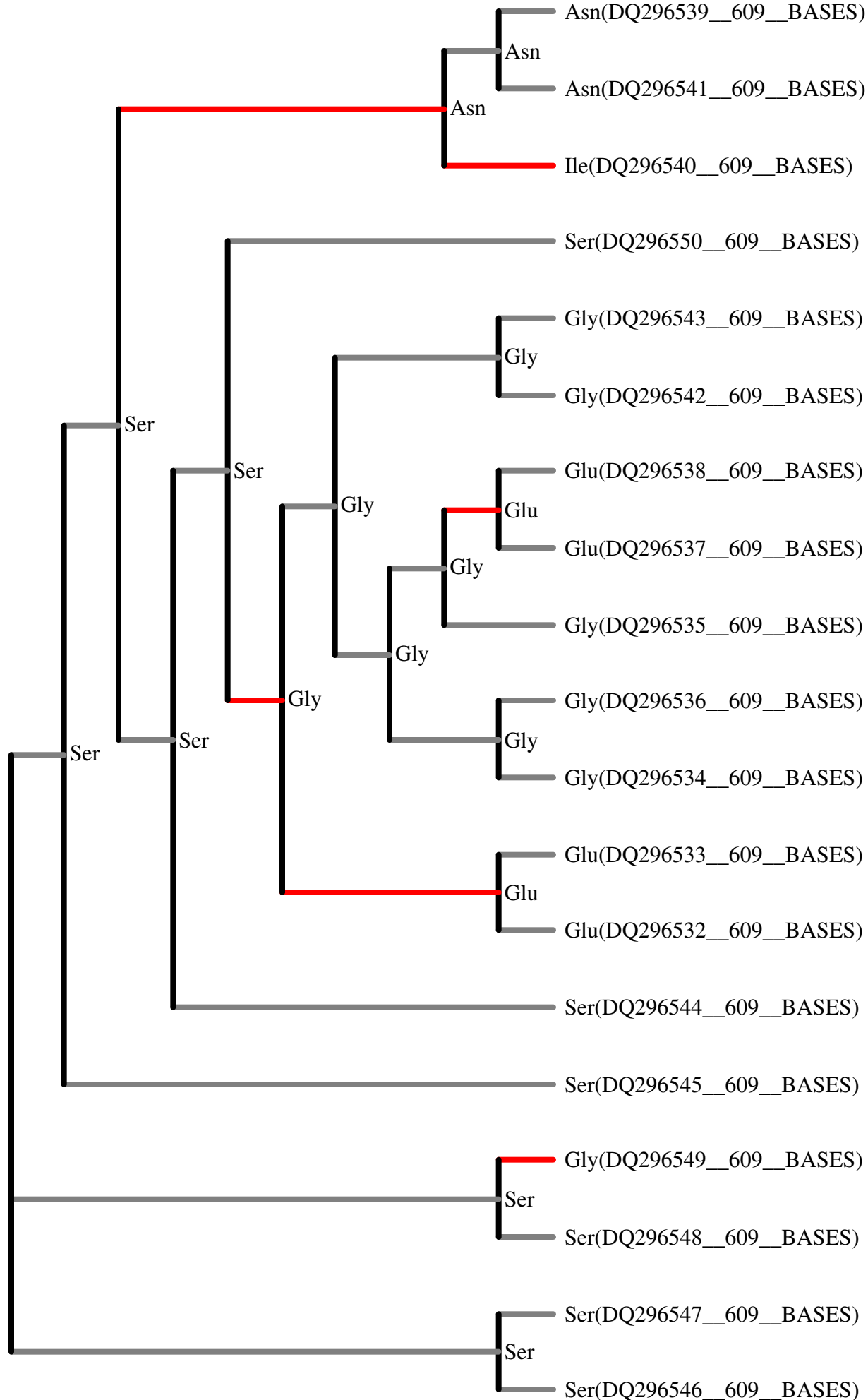

Supplement: Additional file 3 — Ancestral State Reconstruction of the amino acid of site 151 mapped to a phylogeny. Using the Maximum Likelihood the codons ancestral sequence is determined. The arrow indicates the changeover between the both lineages IRN99 and IRN96. [file 1746-6148-2-35-S3.pdf]
